# Alteration of Rumen Bacteria and Protozoa Through Grazing Regime as a Tool to Enhance the Bioactive Fatty Acid Content of Bovine Milk

**DOI:** 10.3389/fmicb.2018.00904

**Published:** 2018-05-08

**Authors:** Melissa L. Bainbridge, Laurel K. Saldinger, John W. Barlow, Juan P. Alvez, Joe Roman, Jana Kraft

**Affiliations:** ^1^Department of Animal and Veterinary Sciences, University of Vermont, Burlington, VT, United States; ^2^Center for Sustainable Agriculture, University of Vermont, Burlington, VT, United States; ^3^Gund Institute for Ecological Economics, University of Vermont, Burlington, VT, United States

**Keywords:** pasture, pearl millet, Illumina MiSeq, Holstein cow, n-3 fatty acids, conjugated linoleic acids, vaccenic acid, odd- and branched-chain fatty acids

## Abstract

Rumen microorganisms are the origin of many bioactive fatty acids (FA) found in ruminant-derived food products. Differences in plant leaf anatomy and chemical composition between cool- and warm-season pastures may alter rumen microorganisms, potentially enhancing the quantity/profile of bioactive FA available for incorporation into milk. The objective of this study was to identify rumen bacteria and protozoa and their cellular FA when cows grazed a warm-season annual, pearl millet (PM), in comparison to a diverse cool-season pasture (CSP). Individual rumen digesta samples were obtained from five Holstein cows in a repeated measures design with 28-day periods. The treatment sequence was PM, CSP, then PM. Microbial DNA was extracted from rumen digesta and sequence reads were produced with Illumina MiSeq. Fatty acids (FA) were identified in rumen bacteria and protozoa using gas-liquid chromatography/mass spectroscopy. Microbial communities shifted in response to grazing regime. Bacteria of the phylum Bacteroidetes were more abundant during PM than CSP (*P* < 0.05), while protozoa of the genus *Eudiplodinium* were more abundant during CSP than PM (*P* < 0.05). Microbial cellular FA profiles differed between treatments. Bacteria and protozoa from cows grazing CSP contained more n-3 FA (*P* < 0.001) and vaccenic acid (*P* < 0.01), but lower proportions of branched-chain FA (*P* < 0.05). Microbial FA correlated with microbial taxa and levels of vaccenic acid, rumenic acid, and α-linolenic acid in milk. In conclusion, grazing regime can potentially be used to alter microbial communities shifting the FA profile of microbial cells, and subsequently, alter the milk FA profile.

## Introduction

Ruminants play a critical role in our food system, converting forages otherwise indigestible to humans, into valuable sources of protein, fat, and other nutrients for human consumption (i.e., meat and milk). Ruminants can utilize forages because of the mutualistic microorganisms (particularly bacteria and protozoa) that reside within their rumen. These rumen microorganisms ferment forage carbohydrates into volatile fatty acids (VFA), which are usable as an energy source by the host animal (Castillo-González et al., 2014). Rumen bacteria and protozoa are also an important source of fatty acids (FA), providing 10–20% of the available lipids to the dairy cow (depending on dietary fat supplementation) (Keeney, 1970). The lipids derived from rumen microorganisms are incorporated into meat and milk products, providing a wide and unique array of bioactive FA.

Among these FA, branched-chain FA (BCFA) are exclusive to the cells of bacteria and regulate the fluidity of their cell membranes (Kaneda, 1991). BCFA possess several human-health benefits, such as anti-cancer activity (Yang et al., 2000; Wongtangtintharn et al., 2004), reducing the risk of necrotizing enterocolitis in newborns (Ran-Ressler et al., 2011), and improving β-cell function (Kraft et al., 2015). Odd-chain FA (OCFA) are produced through bacterial *de novo* lipogenesis using the fermentation product propionate as the substrate (Kaneda, 1991). Blood plasma proportions of OCFA in humans have been linked to a decreased risk of coronary heart disease (CHD) (Khaw et al., 2012) and type 2 diabetes (Forouhi et al., 2014). Rumen bacteria biohydrogenate feed-derived unsaturated FA producing a wide variety of intermediates, such as conjugated linoleic acids (CLA) and vaccenic acid (VA; 18:1 *t*11), that have been shown to reduce tumor growth (Moon, 2014) and risk for CHD (Field et al., 2009). n-3 FA are widely known for their anti-inflammatory, anti-carcinogenic, and cardio-protective effects (Zhao et al., 2004; Liu and Ma, 2014). Typically, n-3 FA are found at low concentrations (<1% of total FA) in dairy products (O'Donnell et al., 2010) because dietary n-3 FA are subject to biohydrogenation by rumen bacteria at a rate of 85–100% (Jenkins et al., 2008), hence, there has been heightened interest in determining approaches to increase the proportion of n-3 FA in dairy products.

Protozoa are less numerous than bacteria in the rumen (10^4^-10^6^ cells/mL vs. 10^10^-10^11^ cells/mL, respectively) (Wright and Klieve, 2011), but because of their larger size (protozoa: 10–200 μm, bacteria: 0.5–2 μm) (Williams and Coleman, 1992), protozoa make up half of the rumen microbial biomass, and thus, supply a large proportion of the microbial FA leaving the rumen (Jouany, 1996). Protozoa facilitate the escape of unsaturated FA from the rumen by engulfing chloroplasts (Huws et al., 2012). Defaunation resulted in a 13% and 10% reduction in the flow of monounsaturated FA (MUFA) and polyunsaturated FA (PUFA), respectively, to the duodenum (Newbold et al., 2015). Therefore, altering the number and type of rumen protozoa and bacteria may be an innovative approach to increase the amount of bioactive FA in ruminant products.

Diet is the main factor driving the shift in rumen microbial communities, and research has demonstrated a change in the number and type of rumen bacteria and/or protozoa in response to supplemental oils (Patra and Yu, 2012), high-grain diets (Fernando et al., 2010), and a switch from total-mixed ration (TMR) to pasture (de Menezes et al., 2011). In addition, Vlaeminck et al. (2006b) demonstrated a shift in the FA composition of bacterial cells with an increasing forage-to-concentrate ratio. Yet, to the best of our knowledge, no studies have evaluated the change in cellular FA that could accompany the shift in microbial communities when cows graze different pasture regimes.

The availability and productivity of summer pastures is a concern for grass-based dairy producers in the Northeast United States. Summer annual pastures (C4 species; e.g., sudangrass, sorgum, and millet) are increasingly popular on grass-based dairy and beef farms, as they grow well during the hot summer months when cool-season perennial pastures (C3 species; e.g., perennial ryegrass, red clover, and orchard grass) experience less growth. Summer annuals generally have more structural carbohydrates (cellulose and hemicellulose) and less PUFA than cool-season species as a result of the differences in plant structure and leaf anatomy (Ball et al., 2001). These differences may favor a different community composition of bacteria and protozoa in the rumen compared to a cool-season pasture (CSP) diet. We have previously shown that feeding dairy cows pearl millet (PM), a summer annual, resulted in a shift in the milk FA profile, particularly increasing the content of BCFA (Bainbridge et al., 2017). Therefore, we hypothesized that grazing dairy cows on (PM), in comparison to a CSP, will shift the rumen microbial community structure (i.e., bacteria and protozoa) and modify their cellular FA composition, resulting in an altered milk FA profile. The objectives of this study were to (i) identify and quantify the rumen bacteria and protozoa when cows graze PM in comparison to a CSP, (ii) evaluate the shift in microbial cellular FA, and (iii) correlate the microbial genera with rumen fermentation parameters, cellular FA composition, and milk FA.

## Materials and methods

### Experimental design

Procedures involving animals were approved by the University of Vermont Animal Care and Use Committee. This research is part of a larger study that has been previously reported (for details see Bainbridge et al., 2017). Briefly, five multiparous (parity: 3.0 ± 0.7 lactations) mid-lactation (171 ± 21 days in milk) Holstein dairy cows were used in a repeated measures design with three 28-day periods. The treatments consisted of two grazing regimes; a diverse CSP and a warm-season monoculture of PM (Table 1). PM was selected for the summer annual because it is palatable, produces high yields, and is drought tolerant. Cows were grazed on CSP and PM for consecutive 28-day periods in the following sequence; PM, CSP, then PM. Cows were supplemented grain twice daily, at each milking, consuming 2.4 kg/day (dry matter (DM) basis) during the entirety of the study (Table 1). Cows consumed all the grain that was supplemented. A 0.5 Ha paddock of CSP and two 0.25 Ha paddocks of PM, seeded in 2-week succession, were used as the forage treatments. These paddocks were subdivided daily to allow for *ad libitum* intake. Cows were rotated to a new paddock subdivision after each milking (2x/day at 630 and 530 h, respectively). All cows had continuous access to water.

**Table 1:** Ingredient and chemical composition (mean ± standard deviation) of the diet components, cool-season pasture (CSP), pearl millet (PM), and grain.

	**Diet component**		
	**CSP**	**PM**	**Grain^a^**
% dry matter (DM)	19.3 ± 2.4	19.9 ± 2.6	89.6 ± 0.6
DM intake, kg/day	14.8 ± 0.7	15.0 ± 1.0	2.42 ± 0.0
**CHEMICAL COMPOSITION, % DM**			
aNDFom^b^	42.5 ± 3.3	53.7 ± 5.0	7.4 ± 1.7
ADF^c^	32.8 ± 2.1	40.5 ± 1.3	10.0 ± 1.4
CP (N x 6.25)^d^	17.4 ± 2.3	14.4 ± 2.3	11.9 ± 1.0
Starch	2.1 ± 0.2	1.2 ± 0.2	30.4 ± 1.1
NFC^e^	24.9 ± 0.7	18.4 ± 1.3	64.1 ± 3.3
Total fatty acids	2.4 ± 0.2	1.3 ± 0.2	2.4 ± 0.1
**FATTY ACID COMPOSITION (MG/G DM)**			
16:0	4.13 ± 0.11	2.93 ± 0.67	4.26 ± 0.41
18:0	0.41 ± 0.01	0.26 ± 0.05	0.56 ± 0.06
18:1 *c*9	0.74 ± 0.10	0.26 ± 0.05	6.63 ± 0.44
18:2 *c*9,*c*12	5.08 ± 0.31	1.83 ± 0.36	10.72 ± 0.96
18:3 *c*9,*c*12,*c*15	12.04 ± 0.96	6.49 ± 0.93	0.69 ± 0.10
∑ other^f^	1.32 ± 0.23	1.16 ± 0.22	0.70 ± 0.09
Total SFA^g^	5.57 ± 0.04	4.10 ± 0.84	5.19 ± 0.49
Total MUFA^h^	0.93 ± 0.13	0.49 ± 0.18	6.89 ± 0.43
Total PUFA^i^	17.17 ± 1.91	8.33 ± 1.26	11.45 ± 1.04
Total n-3 FA	12.07 ± 0.96	6.51 ± 0.94	0.72 ± 0.08
Total n-6 FA	5.13 ± 0.35	1.84 ± 0.36	10.76 ± 0.95

a *The grain consisted of: 47.5% organic corn meal, 16.9% organic whole grain barley, 15.0% organic field peas, 12.5% organic wheat middings, 3.75% calcium carbonate, 1.5% sodium bicarbonate, 1.5% salt, 0.75% kelpmeal, 0.35% magnesium oxide, and 0.25% concentrated base vitamins consisting of: amino acid chelate, manganese amino acid chelate, copper amino acid chelate, vitamin E supplement, selenium yeast, zinc sulfate, zinc hydroxychloride, vitamin A acetate, vitamin D3 supplement, basic copper chloride, sodium selenite, cobalt carbonate, biotin, calcium iodate*.

b *aNDFom, Ash-corrected neutral detergent fiber*.

c *ADF, Acid detergent fiber*.

d *CP, Crude protein*.

e *NFC, Non-fiber carbohydrate = 100 – (NDF + CP + ether extract + ash)*.

f *∑ other: 12:0; 14:0; 15:0; 16:1 c9; 17:0; 18:1 c11; 20:0; 18:3 c6,c9,c12; 20:2 c11,c14; 22:0; 22:1 c13; 20:4 c5,c8,c11,c14; 24:0; 24:1 c15*.

g *SFA, Saturated fatty acids*.

h *MUFA, Monounsaturated fatty acids*.

i *PUFA, Polyunsaturated fatty acids*.

### Forage data and sample collection

An electronic rising plate meter (Jenquip; Feilding, New Zealand) was used to estimate DM intake three times per week as described previously (Bainbridge et al., 2017). Weekly forage samples for quality measurements were collected from the next pasture in the paddock rotation. Each fraction was dried at 65°C to determine DM. Forage samples were ground through a Wiley Mill (Arthur H. Thomas, Philadelphia, PA) with a 2 mm screen and then through an Udy Mill (UDY Corporation; Fort Collins, CO) with a 1 mm screen. Forage samples were analyzed for quality using near-infrared reflectance spectroscopy (Bainbridge et al., 2017).

### Rumen sample collection and processing

On the last day of each period, 1 L of whole rumen digesta was collected via oro-esophageal intubation at 0900 h. On the day of rumen digesta sampling, cows were cut off from feed at 0600 h. Lodge-Ivey et al. (2009) previously demonstrated rumen sampling by oro-esophageal intubation did not differ from sampling via rumen cannula when assessing rumen pH, VFA, and bacterial communities. Sample collection by oro-esophageal intubation of the rumen was selected for this study because it is a tractable method and appears to be a valid alternative (Towne et al., 1990; Santra and Karim, 2002; Cersosimo et al., 2016) to rumen fistula surgery for rumen cannula placement in multiple cows on a commercial dairy farm. Oro-esophageal intubation was also preferred because the cooperating farm was a certified organic dairy, the local certifying agency determined that milk from cows with permanent rumen fistulas could not be marketed as organic. Individual digesta samples were thoroughly mixed and pH recorded (Fisher Scientific Accumet Portable Laboratory pH meter AP110, Pittsburgh, PA). Two aliquots (50 mL) were taken for VFA and microbial identification/quantification, snap frozen in a dry ice and ethanol bath, and stored at −80°C until further analyses. The remaining rumen digesta (900 mL) were used to fractionate rumen bacteria and protozoa by the methods of Or-Rashid et al. (2011) and Lee et al. (2000) with modification by Bainbridge et al. (2016). Briefly, rumen microorganisms were detached from particulate matter using agitation and 1% methylcellulose, then differential centrifugation was used to separate bacterial and protozoal cells. Bacteria and protozoa fractions were confirmed under a microscope to contain <5% feed particulate, and were subsequently lyophilized (FreeZone Plus 2.5, Labconoco, Kansas City, MO) and stored at −20°C.

### FA analyses

Forage FA were analyzed using gas-liquid chromatography according to the method of Bainbridge et al. (2015), VFA were analyzed by gas chromatography (Bainbridge et al., 2016), and microbial FA were analyzed by gas-liquid chromatography/mass spectroscopy (Bainbridge et al., 2016). Milk FA were determined in our previous study by gas-liquid chromatography as described therein (Bainbridge et al., 2017).

### DNA extraction, PCR amplification, and bioinformatics analyses

Microbial DNA was extracted from rumen digesta using the method of Yu and Morrison (2004) with modifications as described by Cersosimo et al. (2014). Bacteria were identified through amplification of the V1-V3 region of the 16S rRNA gene using the bacteria-specific primer pair 27F (5′-AGAGTTTGATCCTGGCTCAG) (Lane, 1991) and 519R (5′-GWATTACCG CGGCKGCTG) (Turner et al., 1999) and protozoa were identified by amplification of the 18S rRNA gene using the protozoal-specific primer pair, P-SSU316F (5′-GCTTTCGWTGGTAGTGTATT-3′) (Sylvester et al., 2004) and GIC758R (5′-CAACTGTCTCTATKAAYCG-3′) (Ishaq and Wright, 2014) as described previously (Cersosimo et al., 2016). PCR amplifications of bacterial DNA were performed under the following conditions: a hot start (98°C for 4 min), followed by 35 cycles of denaturation (98°C for 10 s), annealing (50°C for 30 s), extension (72°C for 30 s), and a 6-min extension in the final cycle. PCR amplifications of protozoal DNA were performed under the following conditions: hot start (94°C for 240 s), followed by 35 cycles of denaturation (94°C for 30 s), annealing (55°C for 30 s), and extension (72°C for 60 s), and final extension of 72°C for 6 min in the last cycle. Molecular Research DNA Laboratories (MRDNA, Shallowater, TX) sequenced the PCR products using Illumina MiSeq v.3. Real-time PCR was used to assess bacterial and protozoal densities and was performed as described by Bainbridge et al. (2016) and Cersosimo et al. (2016), respectively. Bacterial densities are presented using a previously established method; copy number/μL = [measured DNA concentration (ng μL−1)/PCR amplicon length (bp/copy)] × 0.912 × 1012 (Huo et al., 2013). All bioinformatics for 16S and 18S rRNA amplicons were performed in-house by the methods of Bainbridge et al. (2016) and Cersosimo et al. (2016). Briefly, Perl scripts (courtesy of Dr. Benoit St. Pierre, available upon request) were used to screen for quality (>Q30) remove sequences without a forward or reverse primer and bin reads by barcode. The “unique.seqs” command in MOTHUR (v. 1.36.1) was used to determine unique sequences. Conserved regions were aligned using Perl scripts, and the alignment was manually checked. The “chimera.uchime” command was used to remove chimeric sequences and a subsample of 15,000 sequences per sample (based on computing power) was used in the “classify.seqs” command. Sequences were classified down to genus level with an 80% confidence threshold. There were no sequences that did not classify as bacteria or protozoa. Sequence data sets are publicly available through NCBI's Sequence Read Archive, under accession numbers [SRP080847] and [SRP080931].

### Statistical analyses

The PROC MIXED procedure in SAS 9.4 (SAS Institute, Cary, NC) was used to analyze data using a repeated measures ANOVA. Data were first checked for normality using a QQPLOT statement. The statistical model included the fixed effect of diet and the random effect of cow. The fixed effect of diet x period for PM was included in the model and removed if *P* > 0.10. The Kenward-Roger approximation was used for computing the denominator degrees of freedom for the tests of fixed effects resulting from the model. Least-squares (LS) means and standard error (SE) were generated using the LSMEANS/DIFF option to display the results and data were adjusted for multiple comparisons using Bonferroni's method. A power calculation was performed using PROC POWER in the SAS program demonstrating a sufficient power of 0.8 for a two-way ANOVA, with an alpha value of 0.05. Data from the last week of each period were used in statistical analyses (data from CSP and both period 1 and 3 of PM). Significance was declared at *P* < 0.05. Correlation matrices were created using the “cor” function in RStudio, the statistical computing and graphics software (v. 3.3.0), with default parameters (Pearson correlation) and the “corrplot” package using data from the last week of each period across all treatments. Principal Component Analyses (PCAs) were created in RStudio by first, log transforming the data and setting “center” and “scale.” equal to TRUE in the “prcomp” command to standardize the variables prior to preforming the PCA. The PCA was then visualized using the “ggbiplot” function. Data from the CSP treatment from this study were extrapolated to different growing seasons, as the CSP treatment was not repeated.

## Results

### Forage quality and FA composition

The forage quality and FA composition differed between grazing systems (Table 1). There was a higher proportion of ash-corrected neutral detergent fiber (aNDFom) and acid-detergent fiber (ADF) in PM, while CSP contained a higher proportion of protein, starch, and total FA. The FA composition of the grazing regimes varied greatly, CSP contained 2.8-fold more linoleic acid (LA, 18:2 *c*9, *c*12; 5.08 ± 0.31 mg/g DM vs. 1.83 ± 0.36 mg/g DM) and almost 2-fold more α-linolenic acid (ALA, 18:3 *c*9, *c*12, *c*15; 12.04 ± 0.96 mg/g DM vs. 6.49 ± 0.93 mg/g DM) than PM. Overall, CSP contained more total PUFA, total n-6 FA, total n-3 FA, and total MUFA when compared to PM (Table 1).

### Rumen parameters

The total rumen VFA concentration (mM) differed between grazing regimes (Table 2, *P* = 0.029). Although there were no differences in VFA profiles, cows grazed on PM had a higher concentration of VFA than cows grazed a CSP. The pH of rumen digesta did not differ when cows grazed PM vs. CSP (*P* = 0.052; Table 2).

**Table 2:** Rumen parameters [volatile fatty acids (VFA) and pH] from dairy cows^a^ grazing a cool-season pasture (CSP) and pearl millet (PM).

	**Treatment**		**SE**	***P*-value**
	**CSP**	**PM**		
Total VFA (mM)	81.6	101.0	4.96	0.029
**VFA, % TOTAL**				
Acetate	69.6	68.4	0.75	0.39
Propionate	14.9	16.0	1.00	0.53
Butyrate	11.5	10.9	0.49	0.32
Isobutyrate	1.09	1.03	0.02	0.14
Valerate	0.80	0.79	0.03	0.81
Isovalerate	0.61	0.60	0.02	0.82
A:P ratio^b^	4.68	4.43	0.26	0.50
Rumen pH	6.93	6.76	0.05	0.05

a *Least-squares (LS) means are based on n = 5 for CSP and n = 10 for PM*.

b *Acetate:propionate ratio*.

### Rumen protozoal communities

Grazing regime altered the densities of protozoa within the rumen; cows grazed on CSP had higher protozoal densities than cows grazed on PM (4.99 vs. 4.18 log_10_ cells/mL, respectively; *P* = 0.015; Table 3). Sequences were classified into two orders of ciliate protozoa, Entodiniomorphida (averaging 62.17 ± 23.14% across all treatments) and Vestibuliferida (averaging 37.78 ± 21.81% across all treatments), which did not differ by treatment. Regardless of treatment, the most abundant genera within the Entodiniomorphida order were unclassified genera of the Ophryoscolecidae family (averaging 20.02 ± 10.78% across all treatments), *Ostracodinium* (averaging 13.03 ± 8.76% across all treatments), *Entodinium* (averaging 11.55 ± 9.09% across all treatments), *Eudiplodinium* (averaging 10.01 ± 10.39% across all treatments), and *Anoplodinium* (averaging 2.42 ± 1.05% across all treatments). Overall, the most abundant genus within the Vestibuliferida order across all samples was *Dasytricha* (averaging 36.39 ± 15.61% across all treatments) followed by *Isotricha* (averaging 11.43 ± 16.22% across all treatments). Abundance of *Isotricha* was not different when cows grazed PM (24.75%) in comparison to CSP (2.97%; *P* = 0.053; Table 3). Abundance of unclassified genera of the Ophryoscolecidae family was also not different when cows grazed on CSP than on PM (28.91 vs. 15.00% for CSP and PM, respectively; *P* = 0.076). When cows grazed PM, protozoa of the genus *Entodinium* were more abundant within the rumen than when cows grazed CSP (14.05 vs. 1.89% for PM and CSP, respectively; *P* = 0.024). Protozoa of the genus *Eudiplodinium* were more abundant in rumen digesta of cows grazing CSP than PM (29.43 vs. 7.30%, respectively; *P* = 0.022).

**Table 3:** Protozoal communities (% of total sequences) in rumen digesta from dairy cows^a^ grazing a cool-season pasture (CSP) and pearl millet (PM).

	**Treatment**		**SE**	***P*-value**
	**CSP**	**PM**		
Protozoal density^b^	4.99	4.18	0.10	0.015
Entodiniomorphida	72.10	52.36	9.10	0.17
*Anoplodinium*	3.60	1.88	0.74	0.17
*Entodinium*	1.89	14.05	2.48	0.024
*Eudiplodinium*	29.43	7.30	4.57	0.022
*Ostracodinium*	5.25	14.13	4.24	0.25
Un-Ophryoscolecidae^c^	28.91	15.00	4.44	0.076
Vestibuliferida	15.01	42.27	9.62	0.081
*Dasytricha*	14.17	17.51	5.10	0.89
*Isotricha*	2.97	24.75	6.56	0.053
<1% Abundance	11.75	5.37	2.46	0.21

a *Least-squares (LS) means are based on n = 5 for CSP and n = 10 for PM*.

b *Density = log_10_ cells/mL rumen digesta*.

c *Un = unclassified*.

### FA composition of rumen protozoa

The FA composition of rumen protozoal cells was affected by grazing regime (Table 4). Protozoa cells in cows grazing CSP had a higher proportion of VA over cows grazing PM (8.81 vs. 5.54 g/100 g FA, respectively; *P* = 0.005), while a diet of PM resulted in higher total BCFA in protozoal cells (7.13 vs. 5.36 g/100 g FA for PM and CSP, respectively; *P* = 0.011). The n-6 FA, LA, was higher in protozoal cells when cows grazed PM in comparison to CSP (12.54 vs. 7.37 g/100 g FA, respectively; *P* < 0.001) while the n-3 FA, ALA, was nearly 2-fold higher when cows grazed CSP (6.59 vs. 3.54 g/100 g FA for CSP and PM, respectively; *P* < 0.001).

**Table 4:** Fatty acid composition of rumen protozoa in dairy cows^a^ grazing a cool-season pasture (CSP) and pearl millet (PM).

**Fatty acid (g/100 g)**	**Treatment**		**SE**	***P*-value**
	**CSP**	**PM**		
Cyclohexyl-11 11:0	0.08	0.15	0.03	0.12
12:0	0.16	0.13	0.01	0.21
13:0	0.12	0.15	0.01	0.15
*iso* 14:0	0.32	0.33	0.04	0.84
14:0	0.83	0.83	0.03	0.91
14:1 *t*9	0.24	0.32	0.03	0.15
*iso* 15:0	0.50	0.73	0.08	0.15
*anteiso* 15:0	0.92	1.17	0.03	0.008
15:0	1.83	1.98	0.09	0.30
15:1 *t*10	0.13	0.22	0.03	0.10
15:1 *c*10	0.38	0.54	0.05	0.033
*iso* 16:0	1.02	1.59	0.08	0.013
16:0	30.21	35.93	0.41	0.001
16:1 *c*8	0.15	0.12	0.02	0.16
16:1 *c*9	0.82	0.38	0.06	0.006
*iso* 17:0	0.76	0.98	0.08	0.11
*anteiso* 17:0	1.75	2.28	0.09	0.028
17:0	0.63	0.47	0.03	0.023
18:0	23.44	14.49	0.57	<0.001
18:1 *t*4	0.15	0.13	0.01	0.34
18:1 *t*5	0.07	0.07	0.01	0.91
18:1 *t*6-8	0.32	0.24	0.03	0.15
18:1 *t*9	0.25	0.24	0.01	0.78
18:1 *t*10	0.27	0.34	0.04	0.33
18:1 *t*11	8.81	5.54	0.38	0.005
18:1 *t*12	0.37	0.38	0.03	0.76
18:1 *t*13/*t*14	0.64	0.53	0.07	0.36
18:1 *c*9	5.23	9.14	0.29	0.001
18:1 *c*11	0.71	1.00	0.07	0.044
18:1 *c*12	0.15	0.21	0.01	0.033
18:1 *c*14/*t*16	0.30	0.19	0.03	0.058
18:2 *t*10,*t*14	0.24	0.12	0.01	0.001
18:2 *c*9,*t*13/*t8*,*c*12	0.10	0.07	0.01	0.011
18:2 *t*11,*c*15	0.98	0.41	0.04	<0.001
18:2 *t*7,*t*9/*t*10,*t*12	0.06	0.09	0.01	0.16
18:2 *c*9,*t*11	0.43	0.66	0.06	0.082
18:2 *c*9,*c*12	7.37	12.54	0.28	<0.001
18:2 *c*9,*c*11	0.17	0.11	0.02	0.14
18:2 *t*10,*c*12	0.11	0.09	0.01	0.18
18:3 *c*9,*c*12,*c*15	6.59	3.54	0.18	<0.001
19:1 *t*7	0.13	0.12	0.01	0.56
20:0	0.38	0.24	0.02	0.010
20:1 *c*5	0.10	0.07	0.00	0.001
20:1 *c*11	0.05	0.07	0.00	0.029
*iso* 21:0	0.06	0.04	0.01	0.058
22:0	0.36	0.21	0.03	0.015
23:0	0.29	0.93	0.02	0.003
24:0	0.39	0.28	0.03	0.093
Unknown	0.74	0.45	0.04	0.005
Total SFA^b^	58.45	54.80	0.49	0.009
Total MUFA^c^	19.10	19.85	0.37	0.28
Total PUFA^d^	16.12	17.63	0.41	0.067
Total 18:1 *trans*	10.80	7.48	0.29	0.002
Total CLA^e^	0.77	0.95	0.07	0.15
Total n-3 FA^f^	6.59	3.54	0.18	<0.001
Total OCFA^g^	3.54	3.72	0.12	0.75
Total BCFA^h^	5.36	7.13	0.24	0.011

a *Least-squares (LS) means are based n = 5 for CSP and n = 10 for PM*.

b *Total SFA: all saturated fatty acids (12:0–24:0)*.

c *Total MUFA: all monounsaturated fatty acids (14:1–20:1)*.

d *Total PUFA: all polyunsaturated fatty acids (18:2–22:5)*.

e *Total CLA: all detected conjugated linoleic acid isomers: 18:2 c9,t11, 18:2 c9,c11, 18:2 t10,c12, and 18:2 t7,t9/18:2 t10,t12*.

f *Total n-3 FA: The only n-3 present in significant quantities was 18:3 c9,c12,c15*.

g *Total OCFA: all odd-chain fatty acids (7:0–23:0)*.

h *Total BCFA: all branched-chain fatty acids (iso 13:0 to iso 21:0)*.

### Correlations between protozoal communities, VFA, and protozoal FA

**Figure 1:**
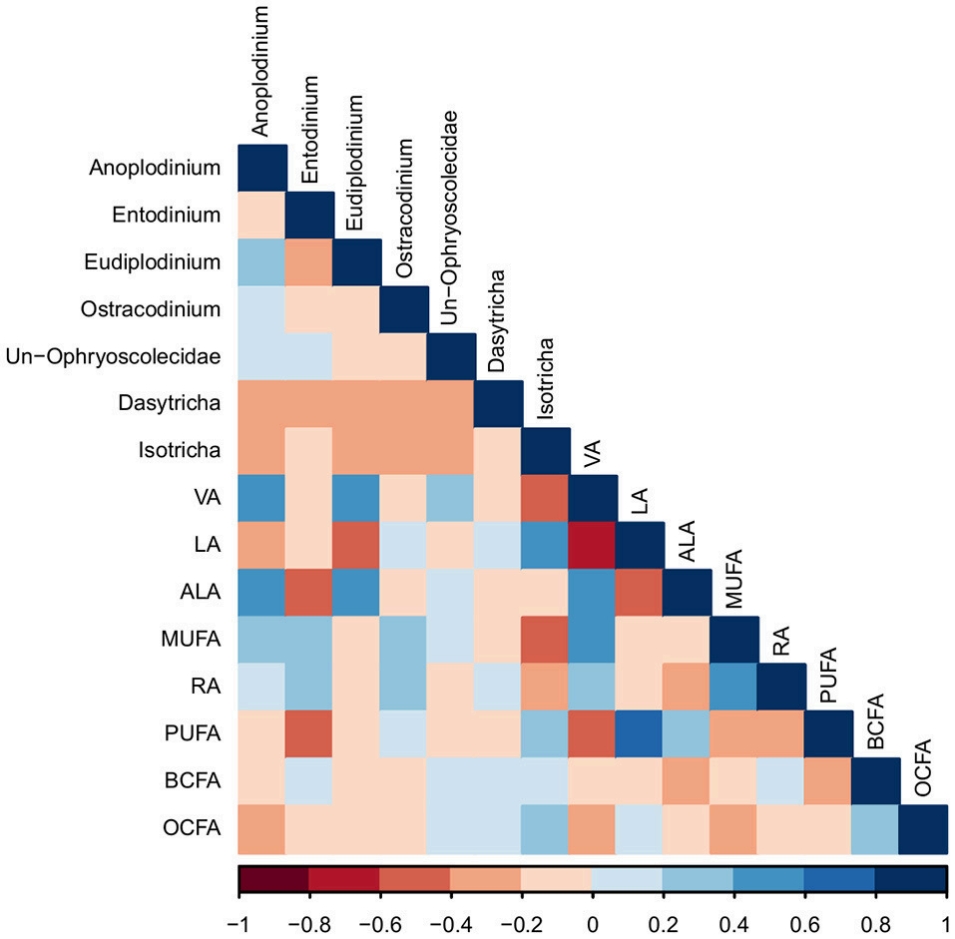
A Pearson correlation matrix between protozoal taxa (>1% abundance) and protozoal fatty acids of cows grazing a cool-season pasture and pearl millet. The scale of the colors is denoted as follows: the more positive the correlation (closer to 1), the darker the shade of blue; the more negative the correlation (closer to −1), the darker the shade of red. Data were used from the last week of each period (*n* = 5 for CSP; *n* = 10 for PM). Un, Unclassified; VA, Vaccenic acid; LA, Linoleic acid; ALA, α-Linolenic acid; MUFA, Monounsaturated fatty acids; RA, = Rumenic acid; PUFA, Polyunsaturated fatty acids; BCFA, Branched-chain fatty acids; OCFA, Odd-chain fatty acids.

The only protozoal genus to correlate with rumen VFA was *Anoplodinium*, which correlated positively with butyric acid (*R* = 0.41; *P* < 0.05; Figure S1). *Dasytricha* were negatively correlated with rumen pH (*R* = −0.39; *P* < 0.05). The proportion of VA in protozoal cells was positively correlated with the genera *Anoplodinium* and *Eudiplodinium* (*R* = 0.50 and 0.45, respectively; *P* < 0.01; Figure 1), while VA in protozoal cells was negatively correlated with the genus *Isotricha* (*R* = −0.52; *P* < 0.01). The proportions of PUFA and ALA in protozoal cells were negatively correlated with protozoa of the genus *Entodinium* (*R* = −0.47 for both; *P* < 0.01), whereas proportions of ALA in protozoal cells were positively correlated with *Anoplodinium* and *Eudiplodinium* (*R* = 0.41; *P* < 0.05, and *R* = 0.52; *P* < 0.01), respectively).

### Rumen bacterial communities

Rumen bacterial densities were greater when cows grazed PM in comparison to CSP (10.20 vs. 9.30 copies/mL rumen digesta; *P* = 0.006; Table 5). Overall, the two predominant bacterial phyla observed in the rumen were Bacteroidetes (averaging 57.58 ± 6.67% across all treatments) and Firmicutes (averaging 37.72 ± 7.49% across all treatments). The only other phylum detected at >1% abundance was Proteobacteria (averaging 1.63 ± 0.61%). Bacteria from the phylum Bacteroidetes were more abundant during PM than CSP (62.24 vs. 52.51%, respectively; *P* = 0.04). The most abundant bacterial genus within the phylum Bacteroidetes, *Prevotella*, was greater in cows grazing PM when compared to CSP (53.87 vs. 40.27%, respectively; *P* = 0.035). Several bacterial genera within the Firmicutes phylum were more abundant during the CSP treatment (41.92 vs. 32.56%, respectively; *P* = 0.045). The genus *Butyrivibrio* constituted 3.56% of the total rumen bacteria when cows grazed CSP, whereas only 1.64% of total bacteria were *Butyrivibrio* when cows grazed PM (*P* = 0.003). The abundance of bacteria belonging to the genera *Coprococcus* and *Roseburia* was greater on CSP (*Coprococcus*: 1.86 vs. 1.32% for CSP and PM, respectively; *P* = 0.034; *Roseburia*: 1.12 vs. 0.70% for CSP and PM, respectively; *P* = 0.047). Unclassified bacteria of the Lachnospiraceae family were more abundant in response to grazing CSP than to PM (6.68 vs. 4.68%, respectively; *P* = 0.029) and unclassified bacteria of the Ruminococcaceae family were also more abundant when cows grazed CSP (7.58 vs. 4.84% for PM and CSP, respectively; *P* = 0.038).

**Table 5:** Bacterial communities (% of total sequences) in rumen digesta from dairy cows^a^ grazing a cool-season pasture (CSP) and pearl millet (PM).

	**Treatment**		**SE**	***P*-value**
	**CSP**	**PM**		
Density^b^	9.30	10.20	0.12	0.006
Bacteroidetes	52.51	62.24	2.15	0.040
*Barnesiella*	1.31	0.87	0.16	0.17
Un-Porphyromonadaceae^c^	1.71	1.31	0.28	0.41
*Prevotella*	40.27	53.87	3.19	0.035
Un-Bacteroidales	4.99	3.33	0.69	0.18
Bacteroidetes <1%	4.20	2.86	0.47	0.045
Firmicuties	41.92	32.56	2.24	0.045
*Butyrivibrio*	3.56	1.64	0.28	0.003
*Coprococcus*	1.86	1.32	0.17	0.034
*Pseudobutyrivibrio*	2.44	1.56	0.27	0.10
*Roseburia*	1.12	0.70	0.12	0.047
Un-Lachnospiraceae	6.68	4.68	0.54	0.029
*Acetivibrio*	1.14	1.12	0.13	0.92
*Ruminococcus*	3.56	3.35	0.35	0.29
Un-Ruminococcaceae	7.58	4.84	0.58	0.038
Un-Clostridiales	4.74	5.40	0.69	0.51
Un-Clostridia	2.70	3.13	0.50	0.47
Fimicutes <1%	6.56	4.84	0.35	0.007
Proteobacteria	1.35	1.84	0.41	0.17
Un-Bacteria	0.47	0.25	0.13	0.079
<1% Abundance	3.74	3.10	0.62	0.57

a *Least-squares (LS) means are based on n = 5 for CSP and n = 10 for PM*.

b *Density = copies/mL rumen digesta*.

c *Un = unclassified*.

### FA composition of rumen bacteria

Cows grazing CSP and PM had differing FA profiles of rumen bacterial cells (Table 6). Total SFA comprised the largest proportion of bacterial cells and were higher when cows grazed PM (78.71 vs. 76.46 g/100 g FA for PM and CSP, respectively; *P* = 0.015). MUFA were the next most prevalent class of bacterial FA and constituted a higher proportion of cells when cows grazed CSP compared to PM (16.78 vs. 15.00 g/100 g FA for CSP and PM, respectively; *P* = 0.029). The proportion of VA was higher in bacterial cells of cows grazing CSP over cows grazing PM (9.09 vs. 5.84 g/100 g FA, respectively; *P* = 0.003). The most notable difference in bacterial FA was seen in BCFA; total BCFA constituted a higher proportion of bacterial cells in cows grazing PM compared to grazing CSP (13.03 vs. 9.70 g/100 g FA for PM and CSP, respectively; *P* = 0.021). The individual BCFA (*aiso* 13:0, *iso* 14:0, *iso* 15:0, *anteiso* 15:0, *iso* 16:0, and *iso* 17:0) were all greater in bacterial cells of cows grazing PM (Table 6).

**Table 6:** Fatty acid composition of rumen bacteria in dairy cows^a^ grazing a cool-season pasture (CSP) and pearl millet (PM).

**Fatty acid (g/100 g)**	**Treatment**		**SE**	***P*-value**
	**CSP**	**PM**		
7:0	0.05	0.08	0.01	0.062
10:0	0.08	0.09	0.01	0.45
11:0	0.03	0.05	0.05	0.14
cyclohexyl-11 11:0	0.28	0.26	0.02	0.44
12:0	0.55	0.58	0.04	0.60
*iso* 13:0	0.37	0.42	0.03	0.33
*aiso* 13:0	0.07	0.11	0.01	0.031
13:0	0.18	0.22	0.01	0.16
*iso* 14:0	0.81	1.11	0.07	0.041
14:0	1.82	2.13	0.17	0.27
14:1 *t*9	0.15	0.24	0.03	0.11
*iso* 15:0	1.59	2.54	0.20	0.026
*anteiso* 15:0	3.86	5.68	0.27	0.015
15:0	2.95	3.87	0.38	0.054
15:1 *c*10	0.30	0.27	0.03	0.53
*iso* 16:0	0.66	0.96	0.06	0.029
16:0	18.44	22.05	0.32	0.002
16:1 *c*8	0.26	0.24	0.03	0.64
16:1 *c*9	1.20	0.82	0.07	0.018
*iso* 17:0	0.41	0.60	0.04	0.016
*anteiso* 17:0	1.51	1.24	0.09	0.13
17:0	0.91	0.97	0.05	0.44
*iso* 18:0	0.06	0.07	0.01	0.73
18:0	40.72	34.07	1.39	0.025
18:1 *t*4	0.10	0.12	0.01	0.13
18:1 *t*5	0.13	0.42	0.07	0.038
18:1 *t*6-8	0.41	0.39	0.03	0.68
18:1 *t*9	0.25	0.24	0.02	0.70
18:1 *t*10	0.45	0.56	0.04	0.068
18:1 *t*11	9.09	5.84	0.36	0.003
18:1 *t*12	0.41	0.51	0.04	0.092
18:1 *t*13/*t*14	1.13	1.18	0.10	0.75
18:1 *c*9	1.08	1.99	0.09	0.001
18:1 *c*11	0.68	0.92	0.07	0.049
18:1 *c*12	0.20	0.36	0.02	0.005
18:1 *c*14/*t*16	0.57	0.64	0.05	0.43
18:1 *c*15	0.14	0.10	0.01	0.064
18:2 *t*10,*t*14	0.39	0.25	0.02	0.013
18:2 *t*11,*c*15	1.19	0.70	0.07	0.006
18:2 *t*10,*c*12	0.19	0.17	0.01	0.39
18:2 *c*9,*t*13/*t8*,*c*12	0.13	0.09	0.01	0.001
18:2 *c*9,*t*11	0.12	0.11	0.02	0.63
18:2 *c*9,*c*12	1.95	2.86	0.15	0.014
18:2 *c*9,*c*11	0.08	0.07	0.01	0.42
18:3 *c*9,*c*12,*c*15	1.49	0.94	0.05	0.001
19:0	0.07	0.07	0.01	0.83
19:1 *t*7	0.09	0.08	0.01	0.78
20:0	0.50	0.57	0.03	0.20
20:2 *c*11,*c*14	0.24	0.30	0.05	0.50
*iso* 21:0	0.02	0.05	0.01	0.041
22:0	0.39	0.44	0.03	0.25
23:0	0.31	0.16	0.01	0.005
24:0	0.38	0.59	0.04	0.017
24:1 *c*15	0.03	0.04	0.01	0.078
Unknown	0.50	0.52	0.05	0.79
Total SFA^b^	76.46	78.71	0.39	0.015
Total MUFA^c^	16.78	15.00	0.37	0.029
Total PUFA^d^	5.78	5.49	0.17	0.33
Total 18:1 *trans*	12.02	9.25	0.39	0.009
Total CLA^e^	0.36	0.35	0.02	0.78
Total n-3 FA^f^	1.49	0.94	0.05	0.001
Total OCFA^g^	4.84	5.69	0.31	0.055
Total BCFA^h^	9.70	13.03	0.58	0.021

a *Least-squares (LS) means are based on n = 5 for CSP and n = 10 for PM*.

b *Total SFA: all saturated fatty acids (7:0–24:0)*.

c *Total MUFA: all monounsaturated fatty acids (14:1–20:1)*.

d *Total PUFA: all polyunsaturated fatty acids (18:2–22:5)*.

e *Total CLA: all detected conjugated linoleic acid isomers: 18:2 c9,t11, 18:2 c9,c11, 18:2 t10,c12, and 18:2 t7,t9/18:2 t10,t12*.

f *Total n-3 FA: The only n-3 present in significant quantities was 18:3 c9,c12,c15*.

g *Total OCFA: all odd-chain fatty acids (7:0–23:0)*.

h *Total BCFA: all branched-chain fatty acids (iso 13:0 to iso 21:0)*.

### Correlations between bacterial communities, VFA, and bacterial FA

**Figure 2:**
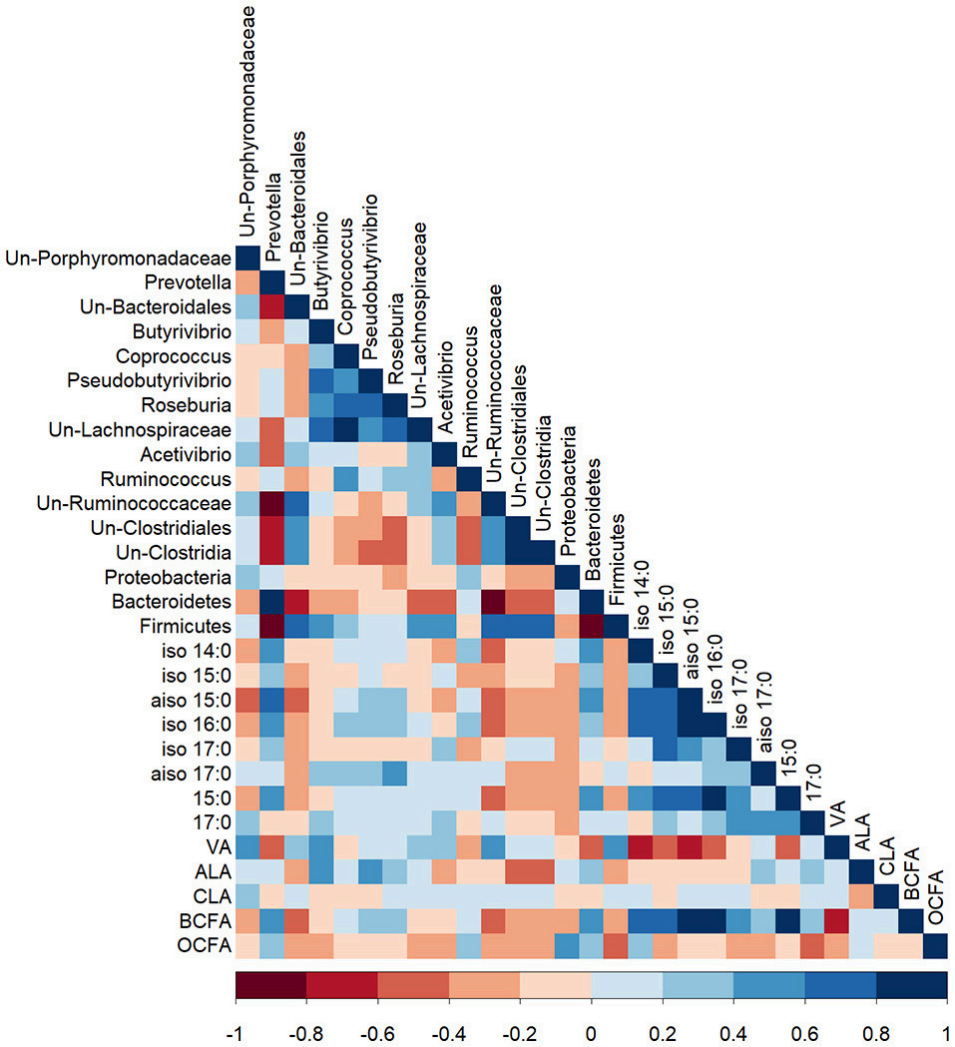
A Pearson correlation matrix between bacterial taxa (>1% abundance) and bacterial fatty acids of cows grazing a cool-season pasture and pearl millet. The scale of the colors is denoted as follows: the more positive the correlation (closer to 1), the darker the shade of blue; the more negative the correlation (closer to −1), the darker the shade of red. Data were used from the last week of each period (*n* = 5 for CSP; *n* = 10 for PM). Un, Unclassified; VA, Vaccenic acid; ALA, α-Linolenic acid; RA, Rumenic acid; BCFA, Branched-chain fatty acids; OCFA, Odd-chain fatty acids.

Bacteria belonging to the genus *Prevotella* correlated positively with the proportion of propionic acid in the rumen (*R* = 0.41; *P* < 0.05) and negatively with rumen pH (*R* = −0.44; *P* < 0.05; Figure S2). There were no other significant correlations between rumen bacteria and rumen parameters (VFA and pH). *iso* 14:0 (*R* = 0.42), *aiso* 15:0 (*R* = 0.53), 15:0 (*R* = 0.47), and total BCFA (*R* > 0.51) were positively correlated with bacteria of the genus *Prevotella* and the phylum Bacteroidetes (*P* < 0.05; Figure 2). Unclassified bacteria of the Ruminococcaceae family were negatively correlated with total BCFA (*R* = −0.53) and individual OBCFA; *iso* 14:0 (*R* = −0.48), *aiso* 15:0 (*R* = −0.57), *iso* 16:0 (*R* = −0.53), and 15:0 (*R* = −0.48) (*P* < 0.01). The proportion of VA in bacterial cells was positively correlated with the abundance of unclassified Porphyromonadaceae (*R* = 0.40), *Butyrivibrio* (*R* = 0.44), unclassified Ruminococcaceae (*R* = 0.48), and total Firmicutes (*R* = 0.46), but negatively correlated with *Prevotella* (*R* = −0.56) and total Bacteroidetes (*R* = −0.55) (*P* < 0.05). The proportion of ALA in bacterial cells was positively correlated with the abundance of *Butyrivibrio* (*R* = 0.44) and *Pseudobutyrivibrio* (*R* = 0.42), and negatively correlated with unclassified bacteria of the class Clostridia (*R* = −0.45) and the order Clostridiales (*R* = −0.42) (*P* < 0.05). OCFA were positively correlated with bacteria from the phylum Proteobacteria (*R* = 0.55) and negatively correlated with bacteria from the phylum Firmicutes (*R* = −0.42; *P* < 0.05).

### Correlations between bacterial and protozoal communities, cellular FA, and milk FA

**Figure 3:**
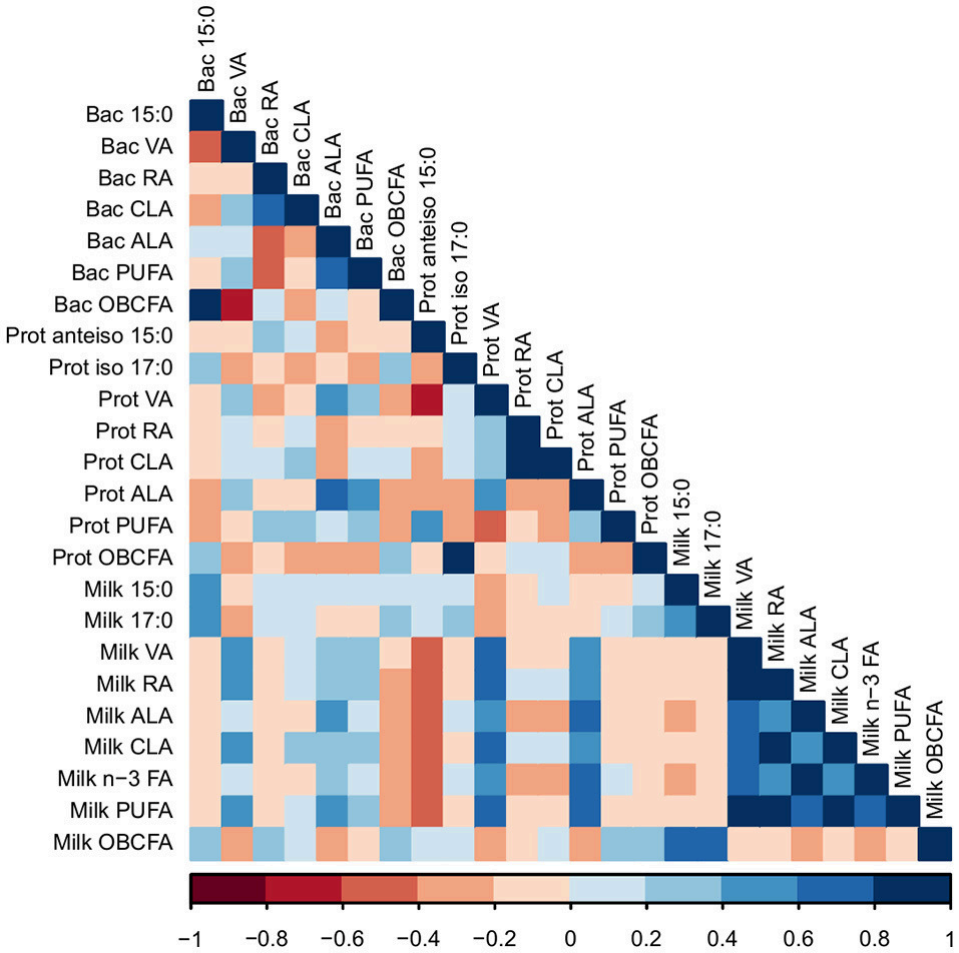
A Pearson correlation matrix between milk fatty acids (FA) and FA in bacterial (Bac) and protozoal (Prot) cells of cows grazing a cool-season pasture and pearl millet. The scale of the colors is denoted as follows: the more positive the correlation (closer to 1), the darker the shade of blue; the more negative the correlation (closer to −1), the darker the shade of red. Data were used from the last week of each period (*n* = 5 for CSP; *n* = 10 for PM). VA, Vaccenic acid; RA, Rumenic acid; CLA, Conjugated linoleic acids; ALA, α-Linolenic acid; PUFA, Polyunsaturated fatty acids; OBCFA, Odd-and-branched-chain fatty acids.

The proportions of 15:0 and 17:0 in milk were positively correlated with the proportion of 15:0 in bacterial cells (*R* = 0.40 and 0.41, respectively; *P* < 0.05; Figure 3). VA in milk was positively correlated with levels of VA and ALA in bacterial cells (*R* = 0.42 and 0.40, respectively; *P* < 0.05). The proportion of milk rumenic acid (RA) was positively correlated with proportion of VA in bacterial cells (*R* = 0.58; *P* < 0.01). The level of ALA in milk was positively correlated to the amount of ALA in bacterial cells (*R* = 0.43; *P* < 0.05). The proportions of RA, VA, CLA, ALA, and total n-3 FA in milk were positively correlated with the proportion of RA in protozoal cells (*R* = 0.61, 0.61, 0.51, 0.62, and 0.46, respectively; *P* < 0.05). Furthermore, the proportions of RA, VA, CLA, ALA, and total n-3 FA in milk were positively correlated with the proportion of ALA in protozoal cells (R = 0.52, 0.46, 0.62, 0.62, and 0.46, respectively; *P* < 0.05). Overall, bioactive FA in bacterial and protozoal cells correlated with bioactive FA in milk fat (Figure 3).

**Figure 4:**
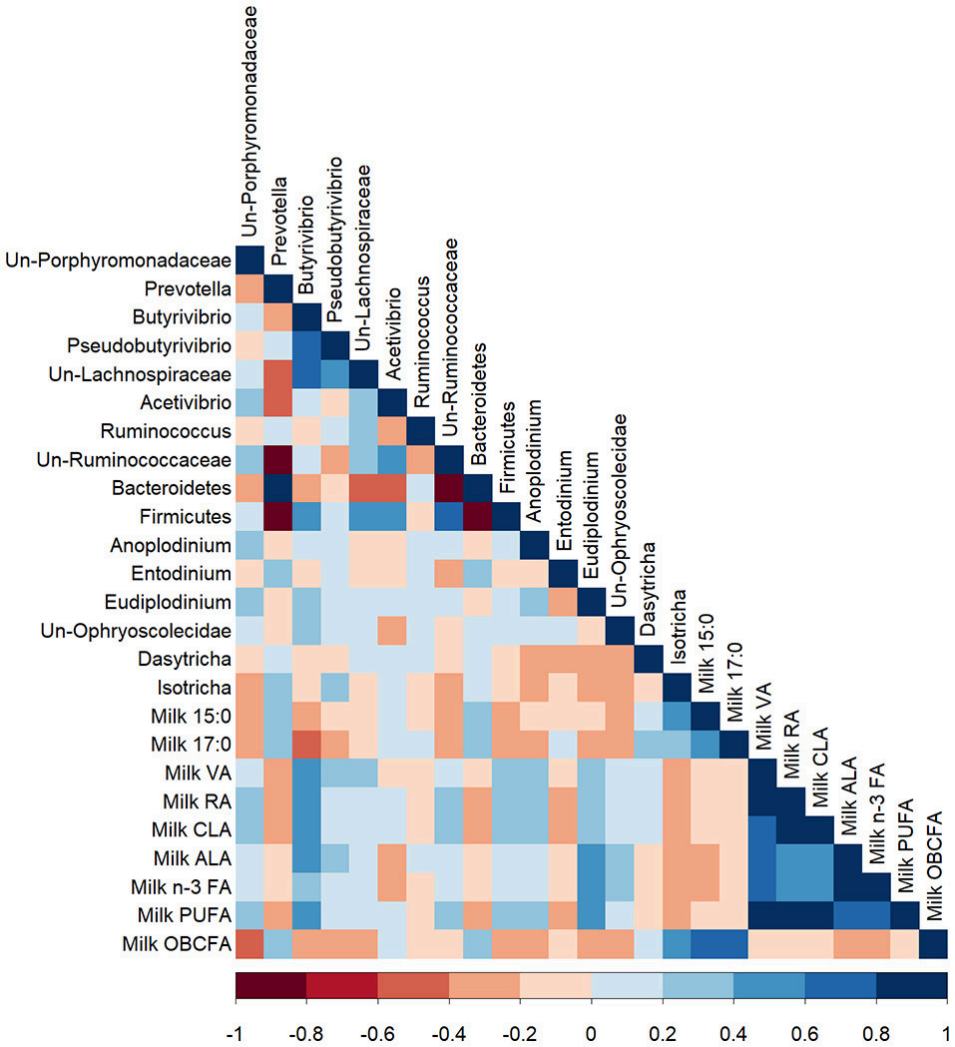
A Pearson correlation matrix between bacterial (Bac) and protozoal (Prot) taxa and milk fatty acids of cows grazing a cool-season pasture and pearl millet. The scale of the colors is denoted as follows: the more positive the correlation (closer to 1), the darker the shade of blue; the more negative the correlation (closer to −1), the darker the shade of red. Data were used from the last week of each period (*n* = 5 for CSP; *n* = 10 for PM). Un, Unclassified; VA, Vaccenic acid; RA, Rumenic acid; CLA, Conjugated linoleic acids; ALA, α-Linolenic acid; PUFA, Polyunsaturated fatty acids; OBCFA, Odd-and-Branched-chain fatty acids.

The proportion of 17:0 in milk was negatively correlated with *Butyrivibrio* (*R* = −0.42; *P* < 0.05; Figure 4). The milk proportions of 15:0 and 17:0 were positively correlated with the bacterial genus, *Prevotella* (*R* = 0.43, and 0.43, respectively; *P* < 0.05), which was confirmed by the PCA (Figure S4). Milk VA, RA, and total CLA positively correlated with bacteria of the genus *Butyrivibrio* (*R* = 0.58, 0.50, 0.47, respectively; *P* < 0.01), which was supported by the PCA. The proportions of milk ALA and total n-3 FA were positively correlated with the bacterial genus *Butyrivibrio* (*R* = 0.41 and 0.39, respectively; *P* < 0.05) and the protozoal genus *Eudiplodinium* (*R* = 0.45 and 0.45, respectively; *P* < 0.01). OBCFA in milk were negatively correlated with unclassified bacteria of the Porphyromonadaceae family (*R* = −0.52; *P* < 0.01), while milk proportion of OBCFA positively correlated with protozoa of the genus *Isotricha* (*R* = 0.44; *P* < 0.05), which was confirmed by the PCA (Figures S3, S4).

PCA of protozoal genera and milk FA demonstrated clustering of variables by treatment, PC1 explained 53.9% of the variation and PC2 explained 18.3% of the variation in the dataset (Figure S3). PCA of bacterial genera and milk FA also demonstrated clustering of variables by treatment, PC1 explained 51.5% of the variation and PC2 explained 15.3% of the variation in the dataset (Figure S4).

## Discussion

Rumen microorganisms synthesize unique FA such as OBCFA, and create biohydrogenation intermediates (e.g., VA and CLA) that are incorporated into milk fat, making it the most distinctive dietary fat in nature. These FA impart beneficial health effects in humans consuming ruminant-derived-food products. Altering microbial communities and their FA metabolism through diet modification can potentially enhance the quantity and profile of these bioactive FA that are available for incorporation into milk and meat. There are currently limited data on how selection of grazing regimen influences the rumen microbial community and, in turn, the milk FA composition. Thus, the aim of this study was to examine the effect of grazing regime, which differed in forage FA composition, on the rumen microbial populations and their cellular FA composition, and correlate these data with the previously reported milk FA profile (Bainbridge et al., 2017).

As shown in previous studies (Or-Rashid et al., 2007; Bainbridge et al., 2016; Cersosimo et al., 2016), the proportion of SFA was higher in bacterial cells than in protozoal cells while the content of unsaturated FA was greater in protozoal cells than in bacterial cells. The proportions of most FA and the differences in FA proportions between bacteria and protozoa observed in the current study were similar to those reported by Or-Rashid et al. (2007) derived from cows fed a TMR. There were, however, a few exceptions; on the one hand, in the current study, the proportions of ALA and 18:1 *trans* isomers in bacterial cells were 4-fold and 3-fold higher, respectively, and the proportion of ALA in rumen protozoal cells was 3-fold higher, presumably as a result of the feeding of fresh pasture in this study. On the other hand, the proportion of total CLA was over 2-fold lower in rumen protozoal cells of the pasture-fed cows in the current study compared to rumen protozoal cells from cows fed TMR (Or-Rashid et al., 2007). Yet, the identity of rumen bacteria and protozoa was not determined by Or-Rashid et al. (2007) and thus, the reason for this discrepancy cannot be explained.

VA is the major 18:1 *trans* isomer formed during the biohydrogenation of dietary PUFA. Ruminant fats are the primary source of VA in the human diet and research demonstrates VA to possess health benefits (Field et al., 2009; Bassett et al., 2010). We have previously demonstrated that the milk proportion of VA is higher when cows graze a CSP in comparison to PM (Bainbridge et al., 2017), and here we further show that the VA proportion in milk is positively correlated with the VA proportion of bacterial cells. The bacterial genus *Butyrivibrio* has been shown to accumulate VA in the rumen (Boeckaert et al., 2008), and the correlation between *Butyrivibrio* species and the proportion of VA in bacterial cells and milk observed in our study is consistent with prior findings. *Butyrivibrio* species preferentially metabolize LA to VA, rather than to other biohydrogenation intermediates (McIntosh et al., 2009). Thus, the higher proportion of LA in the CSP diet combined with the increased abundance of *Butyrivibrio* species in the rumen of cows on CSP may have contributed to the higher VA proportion in microbial cells and consequently in the milk. This study demonstrates that dietary modification may be used to alter bacterial populations and therefore modify biohydrogenation processes resulting in beneficial shifts in the milk FA profile when cows graze a CSP.

Protozoa do not directly participate in the biohydrogenation process (Devillard et al., 2006; Or-Rashid et al., 2008), but they incorporate the biohydrogenation intermediates (e.g., VA and CLA) into their cellular lipids (Devillard et al., 2006). The quantity and flow rate of protozoa from the rumen has been a subject of debate, and a recent review concluded that the flow rate of protozoa is dependent on their type. For instance, holotrich protozoa migrate to the ventral reticulorumen to prevent being transported out of the rumen, while Entodiniomorphids are strongly associated with feed particles and therefore readily leave the rumen with digesta (Newbold et al., 2015). Regardless of species, protozoa account for 20–34% of the total VA and 35–43% of total RA leaving the rumen (Yáñez-Ruiz et al., 2006). It has been hypothesized that this is because of the preferential incorporation of unsaturated FA into their cells to maintain cell fluidity and function (Devillard et al., 2006). Thus, protozoa appear to play a key role in the protection of unsaturated FA in the rumen and contribute to the flow of unsaturated FA to the small intestine (Huws et al., 2012). In this study, an increased proportion of VA in protozoal cells of cows grazing CSP was observed, presumably as a result of the higher amount of LA in the pasture, and hence, increased subsequent biohydrogenation intermediates in the rumen for engulfment by rumen protozoa. Huws et al. (2009) also described higher VA proportions in ruminal protozoa of steers offered fresh grass compared to dry hay. Our study demonstrates that increasing the proportion of VA in protozoal cells correlates with an increased proportion of bioactive FA in milk (e.g., VA, RA, and CLA). Hence, modification of diet (grazing cows on CSP) to increase production of VA by rumen bacteria, in addition to the engulfment and incorporation of this FA by rumen protozoa to prevent further biohydrogenation, may be an effective strategy to achieve greater proportions of bioactive FA in milk.

VA is the primary driver of RA synthesis in the mammary gland through the action of delta-9-desaturase (Griinari et al., 2000). Increasing the availability of VA to the mammary gland increases the proportion of RA in milk. Ruminant products are the largest contributor to RA intake in the human diet (Ritzenthaler et al., 2001), and previous research suggests that RA imparts health benefits (Pariza, 2004; Moon, 2014). Several bacterial species are known to produce RA and VA, including *Butyrivibrio* (Kim, 2003; Wallace et al., 2007), which were identified in this study, and several bacteria, not reported in this study, such as *Bifidobacteria, Lactobacilli*, and *Propionibacteria* (Ogawa et al., 2005). Although multiple bacterial species are involved in the biohydrogenation process, it has been suggested that the main bacteria performing biohydrogenation are cellulolytic (Harfoot and Hazlewood, 1997), potentially explaining the generally higher proportions of RA in ruminant products from pasture-fed cows. In this study, there was no difference between the RA proportion in rumen bacteria when cows grazed CSP and PM, yet, cows grazed on CSP exhibited greater contents of RA in milk (Bainbridge et al., 2017). Hence, it can be confirmed that bacteria contribute to milk proportions of RA through increasing the supply of VA to the mammary gland, which is supported by the positive correlation between the proportion of VA in bacterial cells and the proportion of total RA in milk.

Protozoa may play an important role in the conservation of RA in the rumen as the proportion of RA in rumen fluid was 2-fold higher in faunated vs. defaunated cattle on a TMR diet (Sultana et al., 2011). Yet, we observed no significant correlations between the RA proportion in protozoa and RA proportion of milk. Devillard et al. (2006) determined the FA composition of protozoa isolated from monofaunated sheep and found *Entodinium caudatum* to contain high proportions of CLA. This is similar to the results of the current study, where we observed a trend (*P* = 0.07) toward a positive correlation between *Entodinium* species and the proportion of RA in protozoa. However, this correlation did not persist between the abundance of *Entodinium* and the proportion of RA in milk, presumably because of the overall low abundance of *Entodinium* observed in the rumen. The low concentration of CLA observed in *Isotricha* species by Devillard et al. (2006) was also supported by the negative correlation found between RA and *Isotricha* in the current study. Cersosimo et al. (2016) also observed a positive correlation between *Entodinium* species and the proportion of RA in protozoal cells and a non-significant (*P* = 0.06) negative correlation between *Isotricha* species and RA in three breeds of dairy cow across a lactation. Thus, future research may focus on increasing the abundance of *Entodinium* species in the rumen to potentially enhance the amount of RA in ruminant-derived products.

The proportion of ALA in protozoal cells was positively correlated with the ALA proportion in milk, demonstrating the importance of protozoa to increase the bioactive FA proportion of milk. The higher proportion of ALA in protozoa is purported to be a result of their ability to engulf chloroplasts which contain approximately 60% ALA in their thylakoid membranes (Sandelius and Aronsson, 2009). Huws et al. (2012) noted a higher concentration of ALA in protozoal cells of steers that were fed fresh perennial ryegrass compared to steers fed straw and concentrate. Moreover, the higher proportion of ALA coincided with an increased ingestion of chloroplasts by rumen protozoa (Huws et al., 2012). There is a greater proportion of chloroplasts in leaf tissue than in stem tissue, and as a result of the differences in plant anatomy, cool-season grasses generally have a higher leaf-to-stem ratio than warm-season grasses (Ball et al., 2001). Hence, we hypothesize that CSP provided a higher chloroplast proportion than PM, leading to increased ingestion of chloroplasts by rumen protozoa, which contributed to the 2-fold increase in ALA observed in protozoal cells of cows grazing CSP in comparison to PM. Fluorescence microscopy revealed chloroplasts to be present in 39.7% of protozoa in cows on both hay and fresh grass diets, and only 5.5% of those had greater than 10 chloroplasts per cell (Huws et al., 2009). In particular, the protozoal genera *Epidinium, Polyplastron*, and *Diplodinium* contain more than 10 chloroplasts per cell (Huws et al., 2009, 2012). These genera, however, were observed at lower abundances in the current study than in previous research (Cersosimo et al., 2016), and we speculate that the further enrichments that can be made to the ALA proportion of rumen protozoa from cows grazing CSP if these genera can be increased. A correlation between the protozoal genus *Eudiplodinium* and the proportion of ALA in protozoal cells was observed, which persisted to a correlation between *Eudiplodinium* and the proportion of ALA in milk.

Bacteria synthesize OBCFA *de novo* through the elongation of propionate and valerate in the rumen, or alteration and elongation of α-keto acids, derived from branched-chain amino acids (Kaneda, 1991). As a result, OBCFA are unique to dairy products and are often used as biomarkers for dairy intake in humans (Santaren et al., 2014). In addition, OBCFA are an emerging class of bioactive FA, shown to increase membrane fluidity (Jenkins et al., 2015), reduce tumor growth (Wongtangtintharn et al., 2004), and could be critical to neurological functions, as levels of OCFA were lower in cerebrospinal fluid of patients with Alzheimer's disease (Fonteh et al., 2014). In addition, levels of 15:0 and 17:0 in blood plasma appear to correlate with risk for disease, exhibiting an inverse relationship with development of type 2 diabetes and CVD (Jenkins et al., 2015). The OCFA, 15:0, tended (*P* = 0.054) to be higher in bacterial cells when cows grazed PM and the proportion of 15:0, total BCFA, and total OCFA correlated with the abundance of bacteria in the phylum Bacteroidetes and the genera *Prevotella* within the Bacteroidetes phylum. Other research has previously shown *Prevotella* to be enriched with 15:0 (12.1 g/100 g FA; Vlaeminck et al., 2006a), compared to other bacterial genera, revealing potential for this bacterium to be targeted for modification to enhance the proportion of OCFA in ruminant products. Indeed, the abundance of *Prevotella* was positively correlated with the proportion of 15:0 and 17:0 in milk. Total BCFA and all individual BCFA were higher in the bacterial cells of cows grazing PM. Vlaeminck et al. (2006b) found a higher proportion of BCFA in rumen bacteria when the forage neutral detergent fiber proportion of the diet increased. The PM provided more neutral detergent fiber than CSP, and thus, could presumably be a primary factor driving the greater proportion of BCFA in bacteria, and subsequently milk, from cows on the PM treatment. Bacteria of the genus *Prevotella* were positively correlated with total BCFA, while unclassified bacteria of the Ruminococceae family were negatively correlated with BCFA. These correlations were not observed when we previously evaluated the relationship between bacterial cellular FA and bacterial taxa in three breeds of dairy cows consuming a TMR (Bainbridge et al., 2016), likely as a result of the differing diets in the 2 studies (Bainbridge et al., 2016). Although it has been previously suggested that the OBCFA profile of FA leaving the rumen is more reflective of the abundance of specific bacterial taxa than of available substrate (Saluzzi et al., 1993), our study provides additional evidence that diet is a key factor influencing the bacterial FA profile leaving the rumen.

Understanding the contributions of FA derived from rumen microorganism to the FA composition of ruminant products is central to establishing novel strategies to enhance the proportion of bioactive FA in ruminant milk and meat for human health promotion and maintenance. This study is the first to evaluate the effect of two different grazing regimes on rumen bacterial and protozoal communities and their cellular FA, subsequently affecting the milk FA. We have demonstrated that the interaction between dietary lipids and rumen bacteria produces unique microbial bioactive FA in the rumen and that rumen protozoa have a potential role in preserving these key bioactive FA for incorporation into milk. The higher n-6 and n-3 FA proportion of the CSP led to a substantial increase in n-3 FA and the biohydrogenation intermediate VA in bacterial and protozoal cells. This change was associated with an increase in the milk proportion of VA and n-3 FA of cows on the CSP treatment. Furthermore, the interaction of grazing regime and bacterial communities resulted in a greater proportion of OBCFA in microbial cells of cows grazing PM, which led to an increase in OBCFA in their milk. Based on these data, we conclude there is potential to increase the proportion of bioactive FA in dairy products through management of the diet, which can shift rumen microbial communities and alter FA available to the mammary gland. Future research might focus on tailoring diets to induce shifts in the rumen microbial communities to achieve maximal escape of bioactive FA from the rumen.

## Author contributions

JK, JB, JA, and JR formulated research questions and participated in design of the study. JK, JA, JB, LS, and MB preformed data and sample collection. MB and JK drafted the manuscript and had primary responsibility for the final content. JB, JA, and JR contributed to manuscript revision. All authors read and approved the final manuscript.

### Conflict of interest statement

The authors declare that the research was conducted in the absence of any commercial or financial relationships that could be construed as a potential conflict of interest.
