# Supplementary figures and images for: Alteration of Rumen Bacteria and Protozoa Through Grazing Regime as a Tool to Enhance the Bioactive Fatty Acid Content of Bovine Milk
Source: Front Microbiol. 2018 May 8;9:904. doi: 10.3389/fmicb.2018.00904 (PMC5951984; doi:10.3389/fmicb.2018.00904)

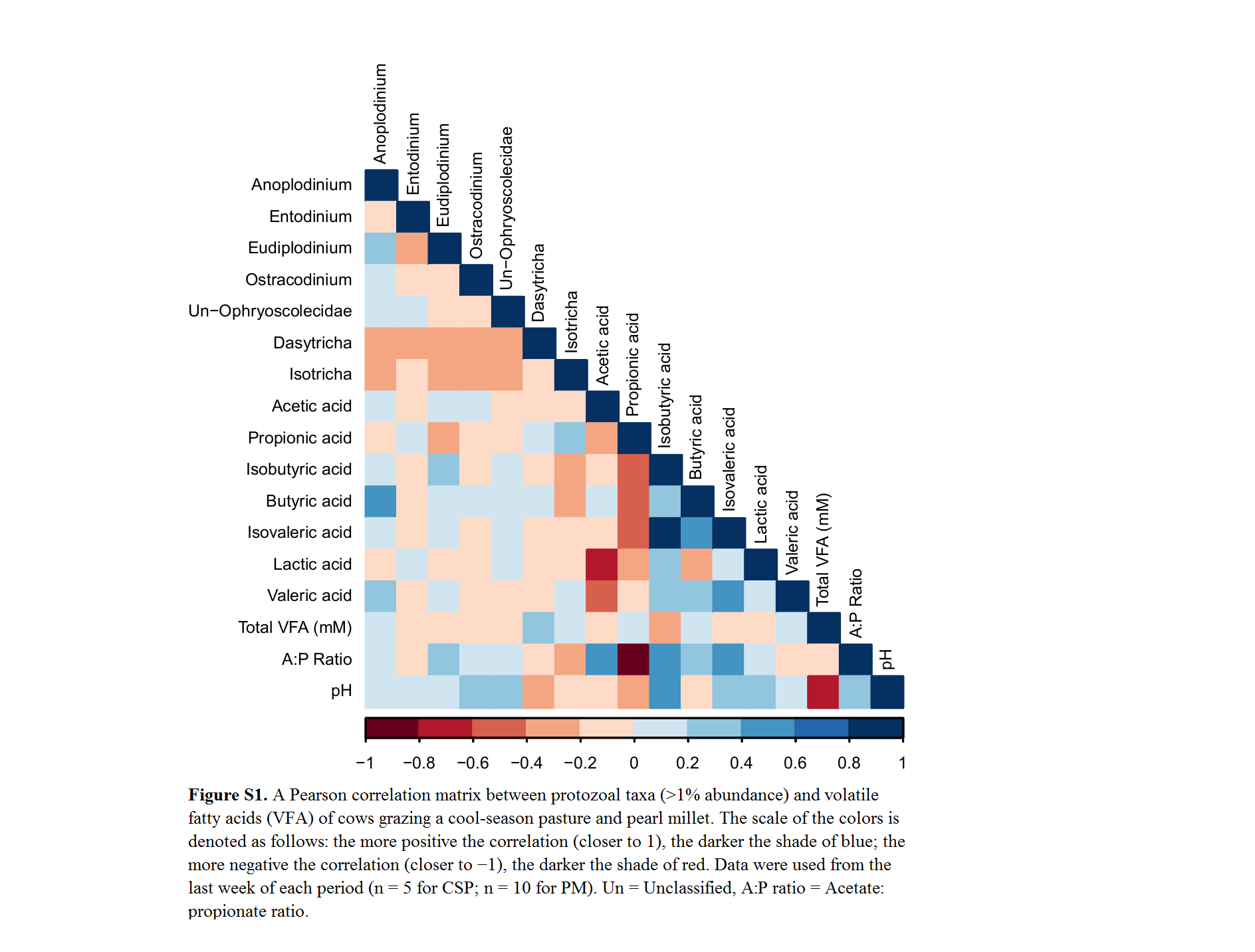

Supplement: Supplementary file 1 [file Image_1.TIF]

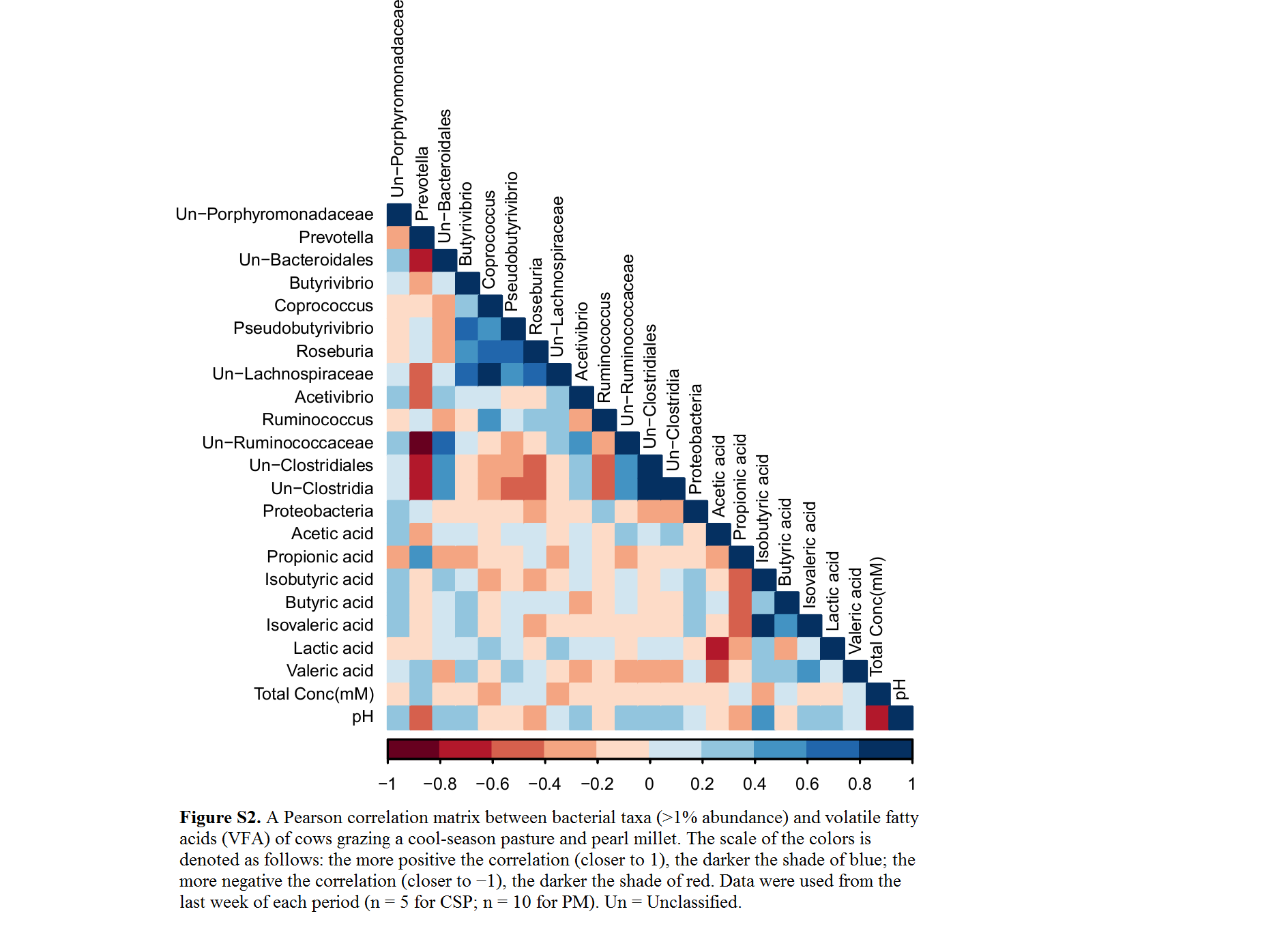

Supplement: Supplementary file 2 [file Image_2.TIF]

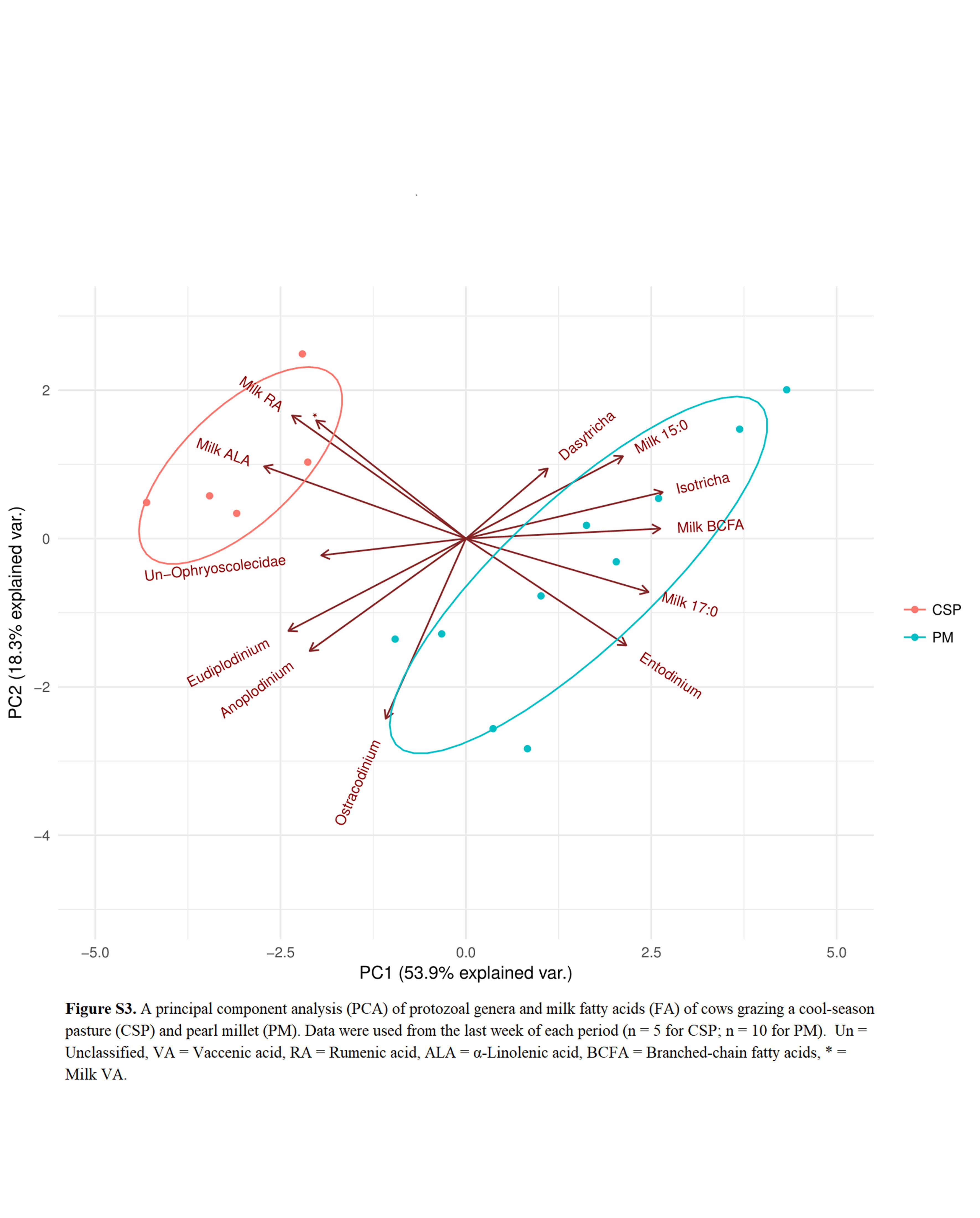

Supplement: Supplementary file 3 [file Image_3.TIFF]

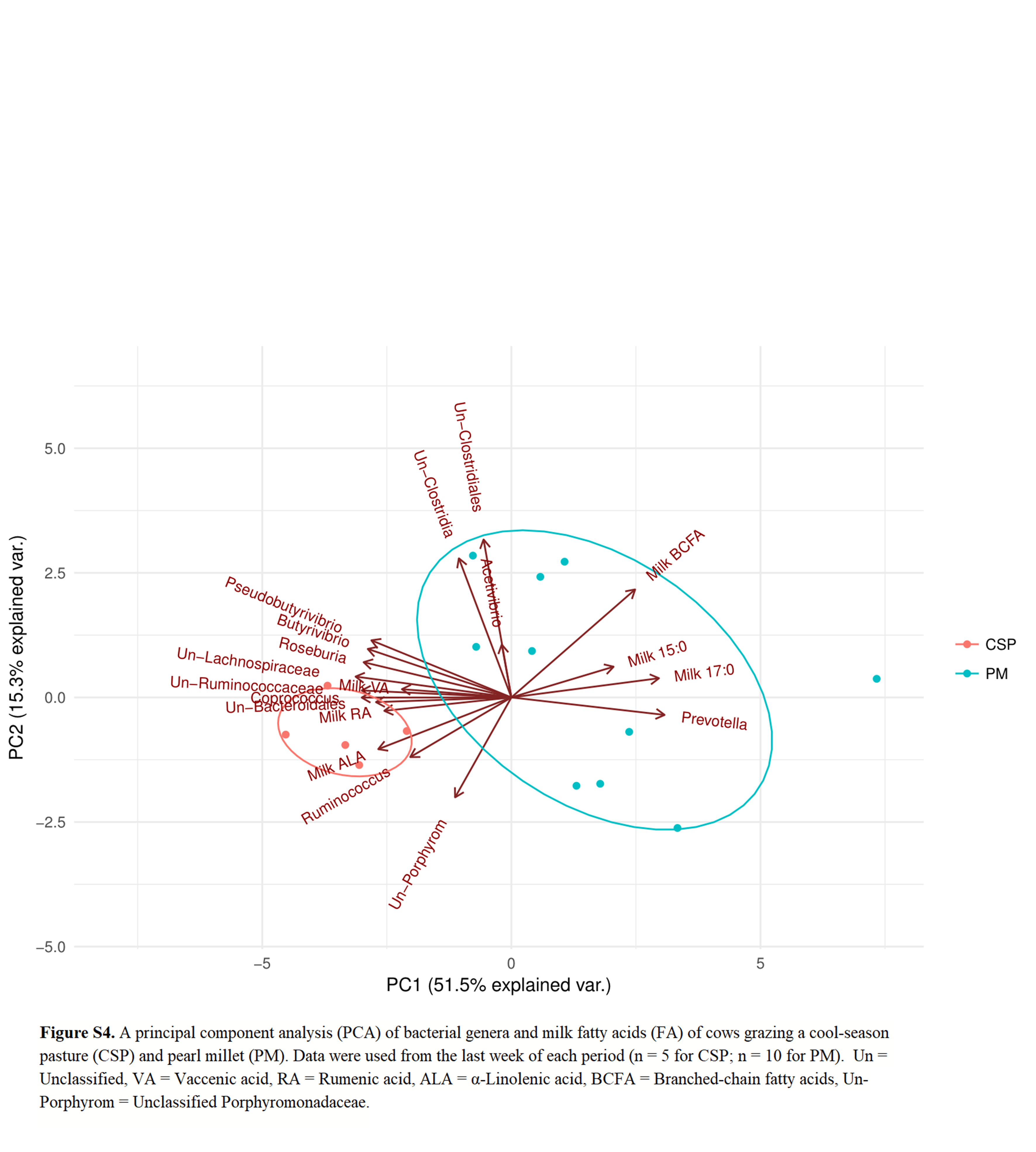

Supplement: Supplementary file 4 [file Image_4.TIFF]
